# Diagnostic Role of Multimodal Imaging in Optic Disc Diseases

**DOI:** 10.3390/diagnostics16162591

**Published:** 2026-08-16

**Authors:** Claudia Fossataro, Maria Cristina Savastano, Francesco Mottola, Francesco De Dominicis, Giorgia Campaniello, Martina Cocuzza, Clara Rizzo, Filippo Amore, Tommaso Salgarello, Andrea Giudiceandrea, Stanislao Rizzo

**Affiliations:** 1 Ophthalmology Unit, Fondazione Policlinico Universitario A. Gemelli IRCCS, 00168 Rome, Italy; 2 Ophthalmology Unit, Catholic University “Sacro Cuore”, 00168 Rome, Italy; 3 National Centre of Service and Research for the Prevention of Blindness and Vision Rehabilitation of the Visually Impaired-IAPB ETS, Fondazione Policlinico Universitario A. Gemelli IRCCS, 00168 Rome, Italy; 4 Department of Neurosciences, Psychology, Drug Research and Child Health, Eye Clinic, University of Florence, AOU Careggi, 50134 Florence, Italy; 5 Consiglio Nazionale delle Ricerche (CNR), 56124 Pisa, Italy

**Keywords:** optic disc, optical coherence tomography, AION, optic disc drusen, glaucoma

## Abstract

The aim of this narrative review was to provide a disease-oriented diagnostic framework for selecting and integrating multimodal imaging techniques according to the suspected optic disc disorder. A non-systematic PubMed search was performed to identify relevant literature on optic disc diseases and related imaging appearances. Searches included a combination of terms related to disease features and imaging modalities. The recent introduction of non-invasive imaging devices, such as optical coherence tomography (OCT) and OCT angiography (OCTA), has allowed a remarkable step forward in the knowledge of the optic disc. OCT has revolutionized the diagnosis and monitoring of glaucomatous eyes, providing useful quantitative biomarkers. Fundus examination and color fundus photography are the first step for evaluation of optic disc edema, which requires further exams, including fundus autofluorescence, to exclude the presence of optic disc drusen, ocular ultrasound and in certain cases, fluorescein angiography. Although still limited, OCTA could give a support in the evaluation of vascular diseases that involve the optic disc, such as anterior ischemic optic neuropathy, retinal vein occlusion and proliferative diabetic retinopathy. Analyzing the radial peripapillary capillary plexus, OCTA can also highlight early vascular changes in degenerative diseases (i.e., glaucoma, Alzheimer’s disease or multiple sclerosis). Multimodal imaging is a cornerstone in the neuro-ophthalmology field, offering unparalleled insight into optic nerve head architecture and vascular integrity in both normal and diseased states.

## 1. Introduction

Accurate assessment of the optic nerve head (ONH) is critical for the diagnosis, classification, and longitudinal monitoring of a wide range of optic neuropathies [1]. The morphology of the ONH—encompassing the optic disc, neuroretinal rim, and peripapillary nerve fiber layer—varies considerably based on demographic, epidemiological, and anatomical factors. Understanding these variations is crucial to distinguish between normal anatomical variants and early pathological changes [2].

Modern advances in ocular imaging have significantly refined our ability to assess the ONH, especially with the introduction of optical coherence tomography (OCT). Structural OCT can provide not only several qualitative structural pieces of information, but also quantitative biomarkers, such as retinal nerve fiber layer (RNFL) thickness and ganglion cell complex (GCC) status [3]. Other structural details can be easily obtained by fundus autofluorescence (FAF), especially in case of suspicious and doubtful optic disc edema, which could sometimes be explained by the presence of optic disc drusen (ODD), also confirmed by OCT and OCT angiography (OCTA) [4,5,6]. Vascular analysis of the ONH is possible by fluorescein angiography (FA), but currently also uses non-invasive OCTA, which allows us to study the radial peripapillary capillary (RPC) plexus in detail. These modalities, combined with OCT, are helpful in the case of ischemic optic neuropathies to make the diagnosis, evaluate the progression and monitor the therapy response [7,8]. Indocyanine green angiography (ICGA) has a marginal role in the evaluation of ONH diseases, with the main potential in inflammatory conditions.

The different imaging modalities allow for precise characterization of ONH anatomy and identification of normal variants such as large physiological cupping, tilted disc and high myopia-related changes, including peripapillary atrophy (PPA), and peripapillary hyperreflective ovoid mass-like structures (PHOMSs), which are best characterized with OCT-based techniques [9]. Rather than reviewing each imaging modality separately, this review adopts a disease-oriented perspective, discussing how multimodal imaging can be integrated to improve diagnostic accuracy and guide clinical decision making across the spectrum of optic disc disorders.

## 2. Imaging

Color fundus photography (CFP) is a fundamental, non-invasive, and cost-effective imaging modality which allows for detailed evaluation and documentation of the optic disc and posterior pole, making it highly suitable for large-scale screening and longitudinal follow-up in ophthalmology [10].

While CFP is highly effective for initial visual assessment, its diagnostic value can be enhanced by incorporating automated image quality assessment [11]. Recent developments in deep learning-based multitask frameworks allow systematic evaluation of CFP quality based on subcategories such as location (centring of ONH or macula), clarity (visibility of fine anatomical structures), and artifact (degree of image obstruction due to opacities or pupil size). By grading images as good, adequate, or poor, these systems help ensure reliability and provide real-time feedback for image acquisition [12]. Notably, such models have achieved high diagnostic accuracy, with reported overall accuracy of 88.15% and Kappa scores of 89.70% on internal datasets [12]. This ensures that CFP images are of sufficient quality for ophthalmologists or artificial intelligence (AI) systems to differentiate subtle features, such as the lumpy-bumpy appearance of ODD versus the diffuse swelling of papilledema, which is vital given the overlapping clinical characteristics which may not be reliably distinguished by clinical history and fundoscopy alone.

Although CFP should be considered additional to in vivo examination of the fundus by indirect ophthalmoscopy, it also allows for reliable comparison over time.

OCT is a high-resolution, non-invasive imaging technique which enables cross-sectional visualization of the retina and ONH based on low-coherence interferometry.

In healthy individuals, OCT of the ONH reveals a well-defined Bruch’s membrane opening, normal RNFL thickness with a double-hump profile (thicker superiorly and inferiorly), a physiological C/D ratio, and preserved neuroretinal rim (Figure S1) [13]. OCT provides useful quantitative and qualitative biomarkers, crucial for the diagnosis of pathological status.

OCTA is a non-invasive imaging modality which enables three-dimensional, depth-resolved visualization of the retinal and choroidal microvasculature by detecting motion contrast from erythrocytes. Considering the ONH, OCTA allows for precise assessment of the RPC plexus, which is not otherwise detectable by FA due to limited resolution and leakage artifacts [14]. In healthy eyes, the RPC plexus appears dense and symmetric, particularly in the superior and inferior sectors aligned with the retinal nerve fibers’ distribution [15].

Since the resolution of this modality, OCTA is particularly valuable in detecting the early microvascular alterations in different optic neuropathies.

Despite its advantages, OCTA has some limitations. Differently from FA, it cannot detect any leakage, making OCTA scarcely useful in case of active exudation. OCTA also suffers from motion and projection artifacts, and lacks universally accepted normative databases due to device-dependent segmentation algorithms [16]. Additionally, considering its recent introduction in everyday clinical practice, interpretation still requires a high level of clinical expertise. Future directions for OCTA include ultra-high-resolution imaging of the lamina cribrosa, integration with AI for automatic segmentation and disease progression tracking, and wider applications in neurology and telemedicine [13].

FAF is a non-invasive imaging technique which enables reconstruction of a monochromatic enface image of the retina and optic disc, using different wave-length light sources [i.e., blue (488 nm) or green (514 nm)]. It captures the excitation of the lipofuscin granules, consisting of bisretinoid, accumulated in the retinal pigment epithelium (RPE); thus, higher lipofuscin density results in greater hyperautofluorescence.

A normal optic disc usually appears hypoautofluorescent due to the absence of the RPE and so do the vessels that obscure the background.

Signal abnormalities develop from abnormal accumulation of lipofuscin, intraretinal or subretinal fluid and loss of RPE.

FA is a useful imaging technique that enables examination of the retinal vascular architecture following intravenous injection of sodium fluorescein. Its wavelength excitation is blue-light (490 nm), while the wavelength emission is green-yellow (520-530 nm). About 50% of fluorescein is bound to plasma, while the free 50% can freely cross the fenestrated choriocapillaris. However, the inner blood–retinal barrier has tight endothelial junctions, which prevent dye diffusion, except in case of damage or pathological neovascularization.

In normal eyes, choroid is the first vascular tissue filled, followed by optic disc and retinal vessels. Since the vascularization of the prelaminar portion of the optic nerve is provided by the posterior ciliary arteries, a mild hyperfluorescence staining of the optic disc appears almost together with the choroidal perfusion [17].

ICGA allows the study of the choroid due to the great affinity (95%) of indocyanine green dye to circulating proteins. Therefore, ICGA is a complementary exam in inflammatory diseases that may involve the choroid and ONH (i.e., granuloma).

## 3. Glaucoma

**Figure 1:**
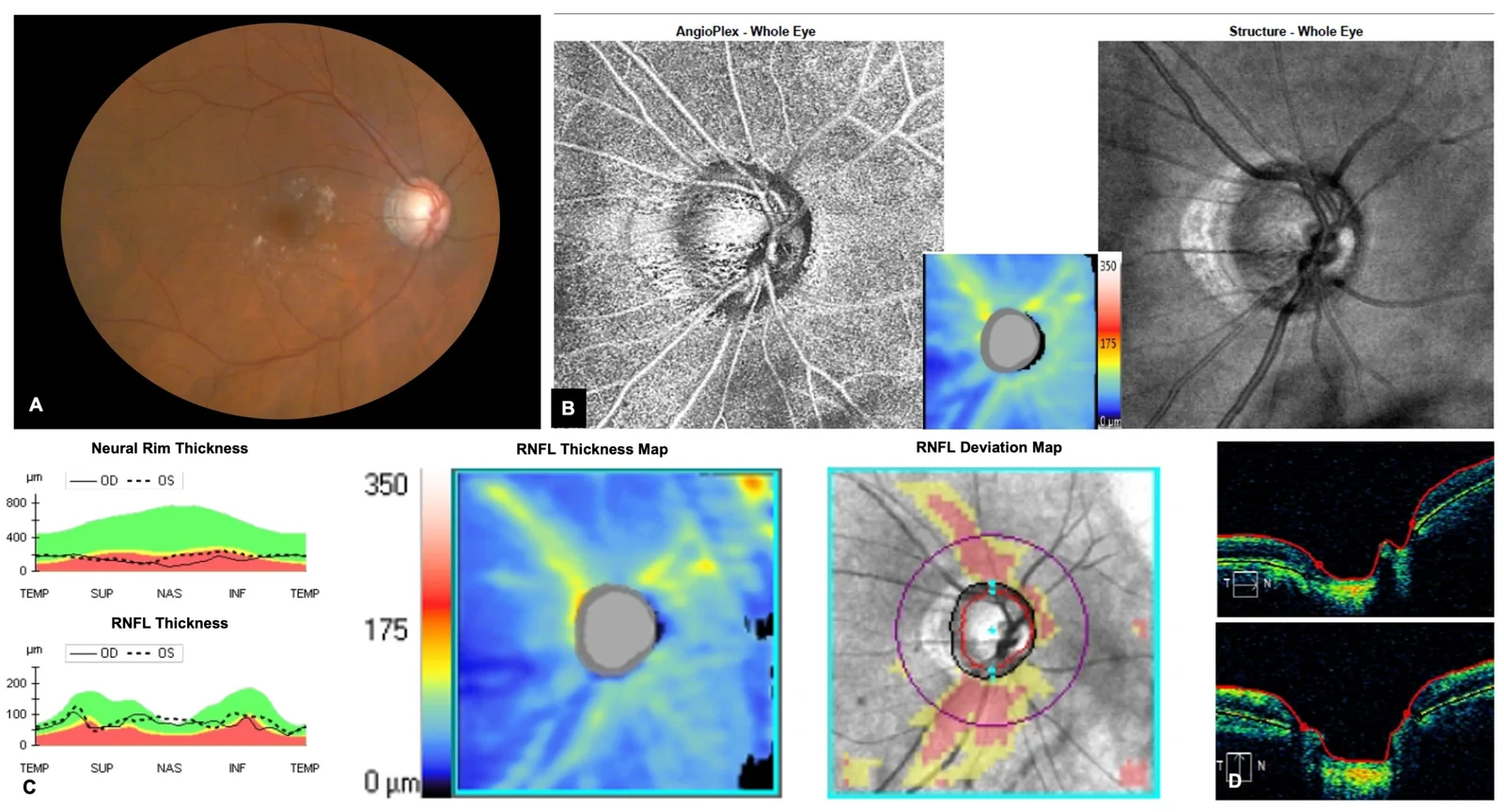
**Glaucoma.** (**A**) The color fundus photography (CFP) shows increased cupping and reduced neural rim. The foveal reflex is preserved, but a yellowish epiretinal membrane is evident in the posterior pole surrounding the macula. (**B**) The optical coherence tomography (OCT) angiography (OCTA) shows significant decreased flow in the radial peripapillary capillary (RPC) plexus, confirmed by the perfusion map. The enface OCT highlights the increased optic disc cupping. (**C**) The neural rim and retinal nerve fiber layer (RNFL) thickness demonstrate a significant decrease in both values, also highlighted in the RNFL Thickness and Deviation maps. (**D**) The horizontal and vertical OCT B scans show the increased cupping.

The first suspicion of glaucoma may arise from the observation of the fundus and the detection of increased optic disc cupping, thinning or notching of the neuroretinal rim [high cup-to-disc (C/D) ratio] or vertical elongation of the cup (Figure 1). Other possible findings include vessel bayoneting, beta-zone peripapillary atrophy and optic disc hemorrhages (DHs), which are considered important markers of glaucomatous progression and are particularly frequent in normal-tension glaucoma (NTG) (Figure S2). Examination of intraocular pressure, visual field test and RNFL analysis by OCT should be recommended in these cases.

In glaucoma, OCT enables early diagnosis, disease staging, and objective monitoring of progression. The most used structural biomarkers include peripapillary RNFL thickness, macular GCIPL (ganglion cell–inner plexiform layer), and the more anatomically accurate BMO-minimum rim width (BMO-MRW). In early and moderate stages, a sectoral or diffuse RNFL thinning is typically observed, particularly in the superior and inferior quadrants, corresponding to visual field defects [18,19]. Moreover, thinning of the macular GCIPL, due to the high density of retinal ganglion cells in the central retina, may precede peripapillary RNFL thinning and provide earlier detection of damage (Figure 1) [20].

BMO-MRW, a parameter derived from 3D scans of the ONH, has been shown to correlate better with functional loss than traditional measurements (i.e., vertical C/D ratio or rim area), especially in myopic eyes or in those with small optic disc [19]. These parameters also help differentiate true glaucomatous changes from physiological cupping or congenital anomalies.

Particular attention should be paid to NTG, in which structural damage may occur despite statistically normal intraocular pressure values. OCT frequently demonstrates localized RNFL and GCIPL thinning, particularly involving the inferotemporal and superotemporal sectors. Furthermore, optic DHs are more frequently observed in NTG than in high-pressure glaucoma and are considered important predictors of disease progression, often preceding measurable structural and functional deterioration.

OCT is a fundamental modality to monitor the glaucoma progression. In early stages, structural parameters (RNFL and GCIPL) indeed are more sensitive than functional tests. However, in advanced glaucoma, these metrics tend to reach a “floor effect,” limiting their utility. In such cases, functional assessment with standard automated perimetry becomes more informative. Combining OCT with visual field tests, using guided progression analysis (GPA) or AI-based software, enhances the ability to detect subtle changes over time.

Longitudinal OCT assessment plays a pivotal role in glaucoma management. Serial measurements of RNFL, GCIPL and BMO-MRW may identify progressive tissue loss before clinically significant visual field deterioration becomes evident. Modern OCT platforms provide both event-based and trend-based analyses, allowing the quantification of structural progression and improving the risk stratification.

Emerging evidence suggests that mitochondrial dysfunction may contribute to retinal ganglion cell degeneration in glaucoma. Structural OCT studies indicate that metabolic impairment may precede measurable tissue loss, supporting the hypothesis that mitochondrial abnormalities play an important role in glaucomatous neurodegeneration and disease progression [21].

In glaucomatous eyes, OCTA reveals focal or diffuse reductions in RPC vessel density (VD), even before structural defects become evident in OCT RNFL and visual field exam. Jia et al. demonstrated that microvascular perfusion in the ONH was significantly reduced in early glaucoma and strongly correlated with visual field defects, independently from the rim loss or the C/D ratio [22,23]. VD and perfusion density (PD) represent the two most commonly used quantitative parameters to study the RPC plexus, offering high sensitivity in early disease detection [24].

In summary, the examination of the ONH by indirect ophthalmoscopy, supported by CFP, is necessary to raise the suspicion of glaucoma as well as to follow up already diagnosed patients. The analysis of RNFL and GCIPL by OCT and visual field test represent the first-line tests to diagnose and monitor the progression or stability of the disease, in association with intraocular pressure (IOP) measurement. Nowadays, OCTA is a complementary exam in glaucomatous eyes, although its significance is increasing, considering the chance to detect very early changes in ONH vascularization. Further tests will be required only in doubtful cases to make differential diagnosis.

## 4. Optic Disc Drusen

A comprehensive evaluation and in-depth understanding of optic disc morphology are essential for accurately differentiating among various causes of ONH elevation, such as ODD, papilledema, and other forms of optic disc edema (ODE). A non-pathological condition such as the tilting disc can mimic disc edema on CFP due to elevation of the superotemporal rim and blurred margins. However, this anomaly is generally benign, non-progressive, and typically asymptomatic [25].

ODD, which are acellular, often calcified, mucoprotein deposits, can simulate disc swelling, defined as pseudopapilledema. These drusen are believed to arise from impaired axonal transport that leads to axonal rupture and release of mitochondria, which subsequently act as a nidus for drusen formation [26].

Recent studies have further suggested that mitochondrial accumulation and dysfunction may contribute to the pathogenesis of ODD. Altered axonal metabolism and mitochondrial stress have been proposed as mechanisms underlying the ganglion cell dysfunction and some structural changes detectable by OCT [13].

**Figure 2:**
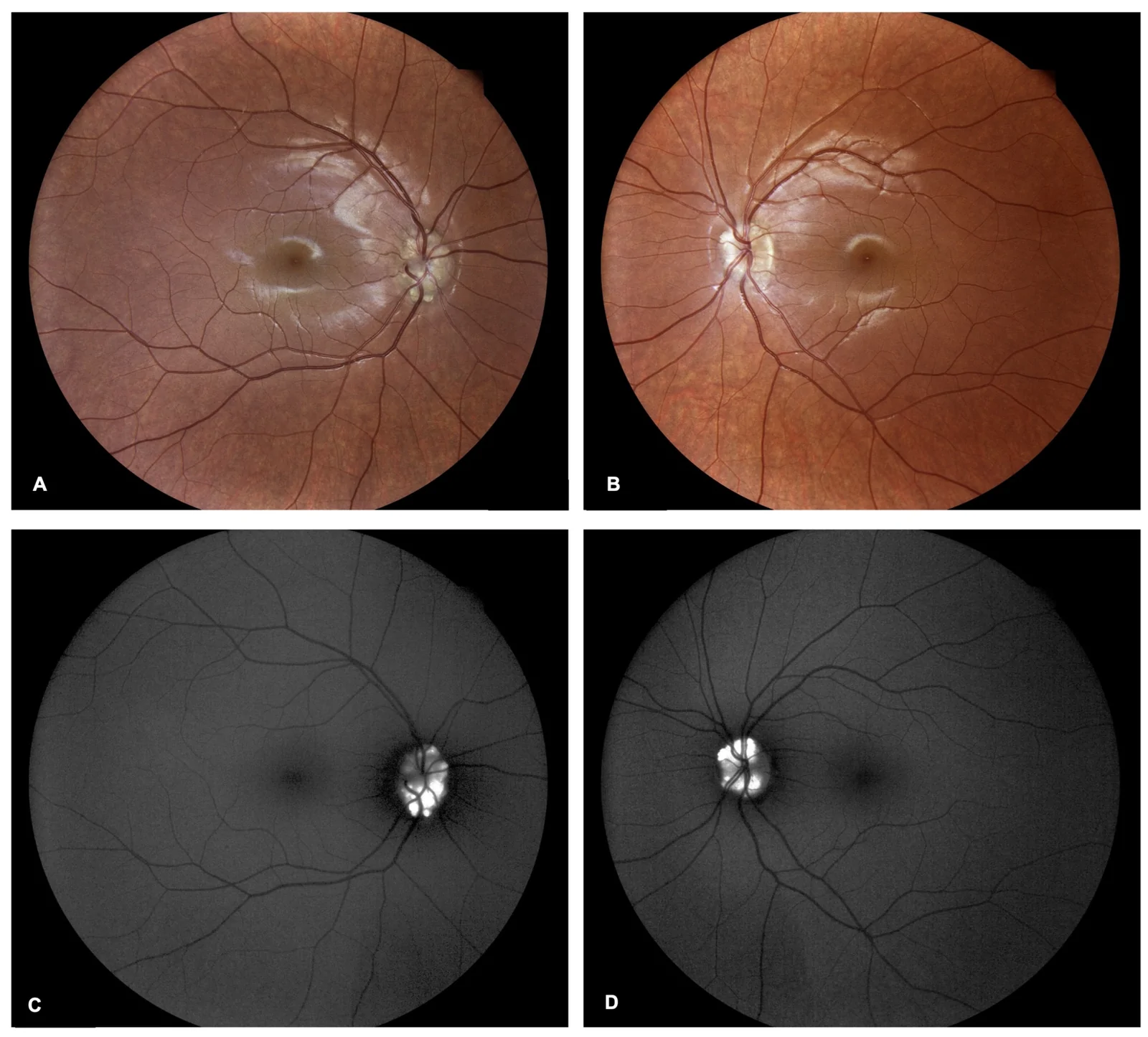
**Optic disc drusen (ODD).** (**A**,**B**) Color fundus photography (CFP) shows yellowish optic deposits in both eyes (OD > OS). (**C**,**D**) Fundus autofluorescence reveals the presence of ODD, which appear hyperautofluorescent.

ODD are usually bilateral and occur in approximately 2% of the general population. They are typically present at birth or during early childhood and can increase in size and number during adolescence, gradually migrating from deeper to more superficial layers with age [27]. On CFP, visible ODD may appear as yellowish, highly refractile bodies, contributing to a crowded, lumpy-bumpy appearance of the optic disc, with blurred margins and from very small to absent C/D ratio (Figure 2) [28]. However, deeper ODD may not always be visible by ophthalmoscopy or CFP, complicating the differentiation from a true disc edema. This distinction is critical, as ODD are generally benign but may be associated with visual loss, particularly in the context of non-arteritic anterior ischemic optic neuropathy (NAION) [29].

FAF plays a crucial role in the study of ODD, which appear hyperautofluorescent (Figure 2) [28]. The intensity correlates with drusen size and depth in the ONH. If superficial, they can be easily detected by fundus examination and FAF, which has a high sensitivity for their recognition. On the other hand, deep drusen, also known as buried drusen, are not always revealed by FAF, especially if located below the inner limiting membrane (ILM), more than 500 µm [30].

Furthermore, ODD are often associated with abnormal vasculature patterns like vessel segmentation, tortuosity and collaterals. FAF can easily study these conditions, taking advantage of the hypofluorescent signal of the blood [31].

FAF could support the differential diagnosis between ODD and ODE, highlighting in the latter the indistinct hypoautofluorescent margins of the optic disc. However, to definitely exclude an ODE, standardized ocular ultrasound B and A scans, in association with magnetic resonance imaging (MRI), are recommended.

OCT B-scan is particularly effective in detecting ODD, which appear as hyperreflective sub-surface deposits often accompanied by OCT RNFL irregularities. This imaging modality, particularly using the EDI tool, with FAF and optic disc ultrasound, is crucial to make the differential diagnosis with true papilledema.

Fundus examination and CFP could be sufficient to make the diagnosis of ODD; however, it is advisable to confirm their presence by FAF and ocular ultrasound, at least at their first detection. Structural OCT and RNFL analysis provide significant support to confirm the diagnosis. OCTA and FA should be required only in doubtful cases.

## 5. Optic Disc Edema

In case of swollen optic disc with indistinct margin, the differential diagnosis between the possible causes of ODE could be reached by the integration of imaging modalities.

Papilledema refers specifically to ODE resulting from increased intracranial pressure (ICP). At CFP, papilledema presents as a swollen, elevated optic disc with blurred margins, and it is often accompanied by venous dilation, peripapillary hemorrhages, exudates, and cotton wool spots in acute stages [32]. Its severity can be assessed using grading systems such as the modified Frisén scale, which describes nerve fiber layer changes from minimal (Stage 1, subtle C-shaped halo) to severe ones (Stage 5, complete obscuration of vessels on and leaving the disc) [33]. Although typically bilateral, papilledema can be asymmetric, and clinical symptoms often include headache and transient visual obscuration. If left untreated, papilledema may progress to optic atrophy and irreversible vision loss, even after the disc swelling resolves [4].

**Figure 3:**
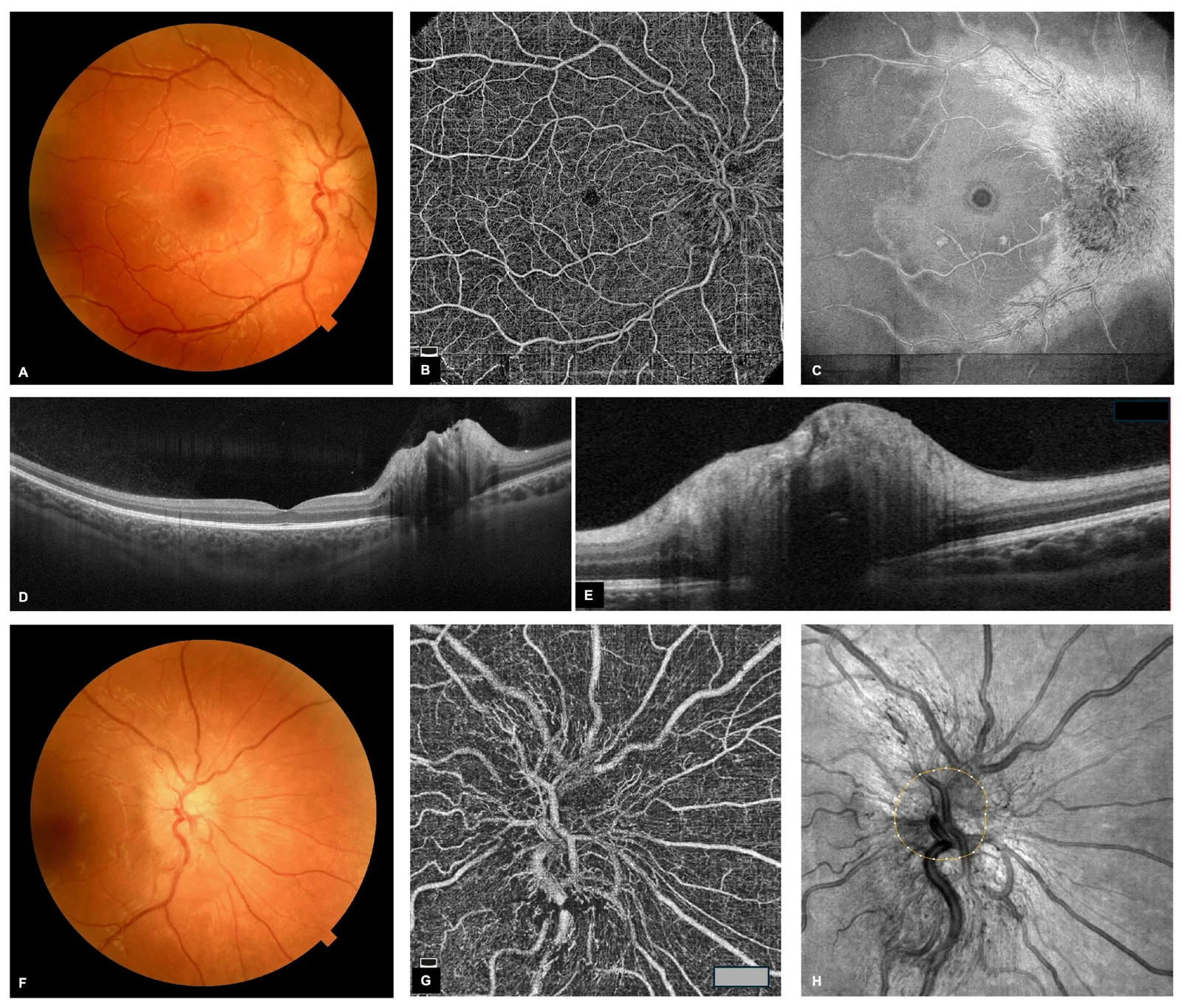
**Optic disc edema.** (**A**,**F**) Color fundus photography shows optic disc swelling, indistinct margins and few flamed hemorrhages. (**B**,**G**) Optical coherence tomography (OCT) angiography (OCTA) shows the flow deficit in the radial peripapillary capillary (RPC) plexus. (**C**,**H**) Enface OCT scans reveal the indistinct margins of the optic disc. (**D**,**E**) OCT B scan shows the swelling of the optic disc, with preserved macular structure, absence of intraretinal or subretinal fluid.

**Figure 4:**
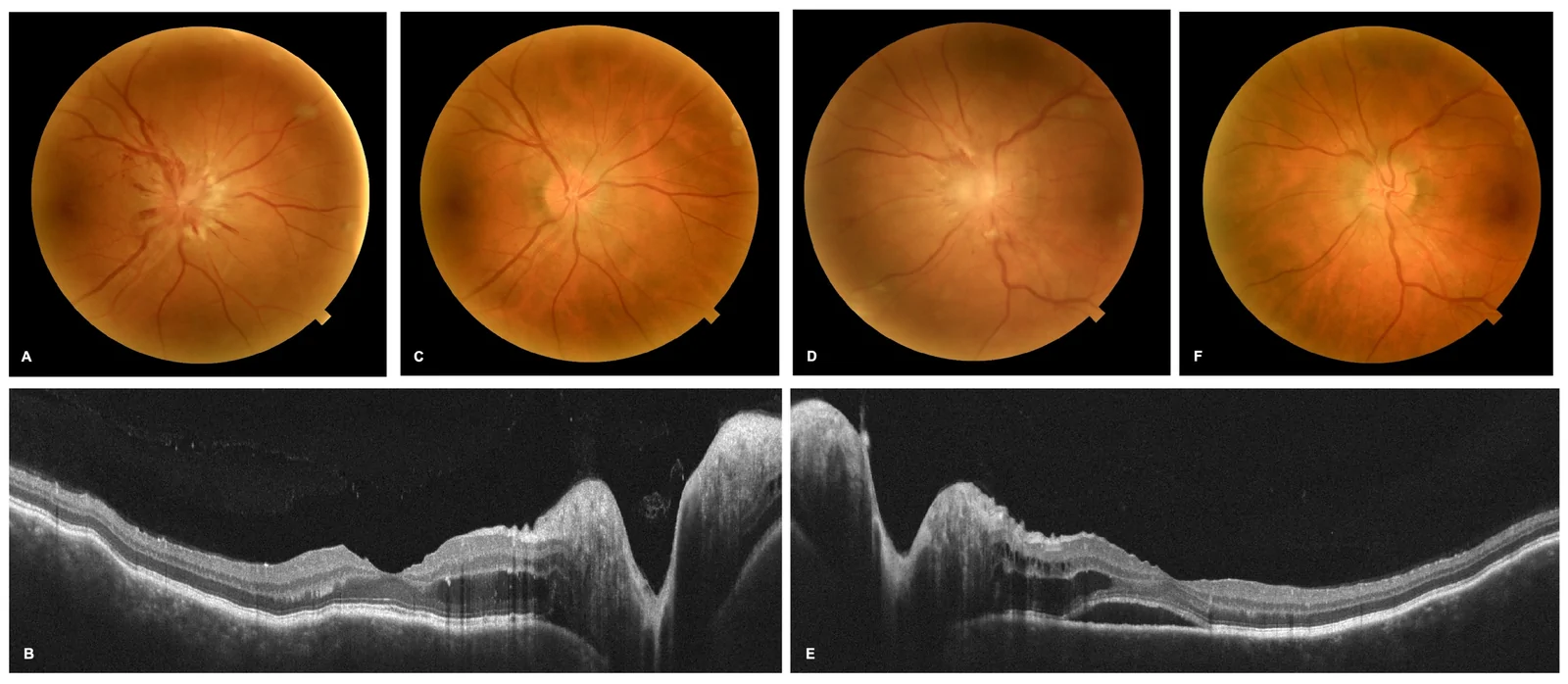
**Bilateral optic disc edema.** (**A**) Color fundus photography (CFP) of the right eye shows optic disc swelling, flamed hemorrhages and choroidal folds. (**B**) Optical coherence tomography (OCT) confirms the optic disc swelling and shows intraretinal fluid and choroidal folds. (**C**) CFP of the right eye, 40 days after treatment, shows recovery of the optic disc, with distinct margins and reabsorption of the hemorrhages. (**D**) CFP of the left eye in the acute phase shows swelling of the optic disc, choroidal folds and flamed hemorrhages. (**E**) OCT shows intraretinal and subretinal fluids, only slight choroidal folds, and confirms the optic disc edema. (**F**) CFP of the left eye shows complete recovery 40 days after treatment.

Among the other causes of ODE, anterior ischemic optic neuropathy (AION), including both NAION and arteritic (AAION), often presents with reduced visual acuity, color vision impairment and visual field defects. In CFP images, NAION commonly appears as sectoral disc edema (e.g., inferotemporal), while diffuse pallid disc edema may suggest an arteritic cause (Figure 3 and Figure 4). When NAION occurs in patients with ODD, CFP may reveal superimposed disc edema and hemorrhages overlying the drusen [34]. It is hypothesized that ODD, causing structural crowding of the ONH, may independently increase the risk of NAION, especially in younger individuals [35].

**Figure 5:**
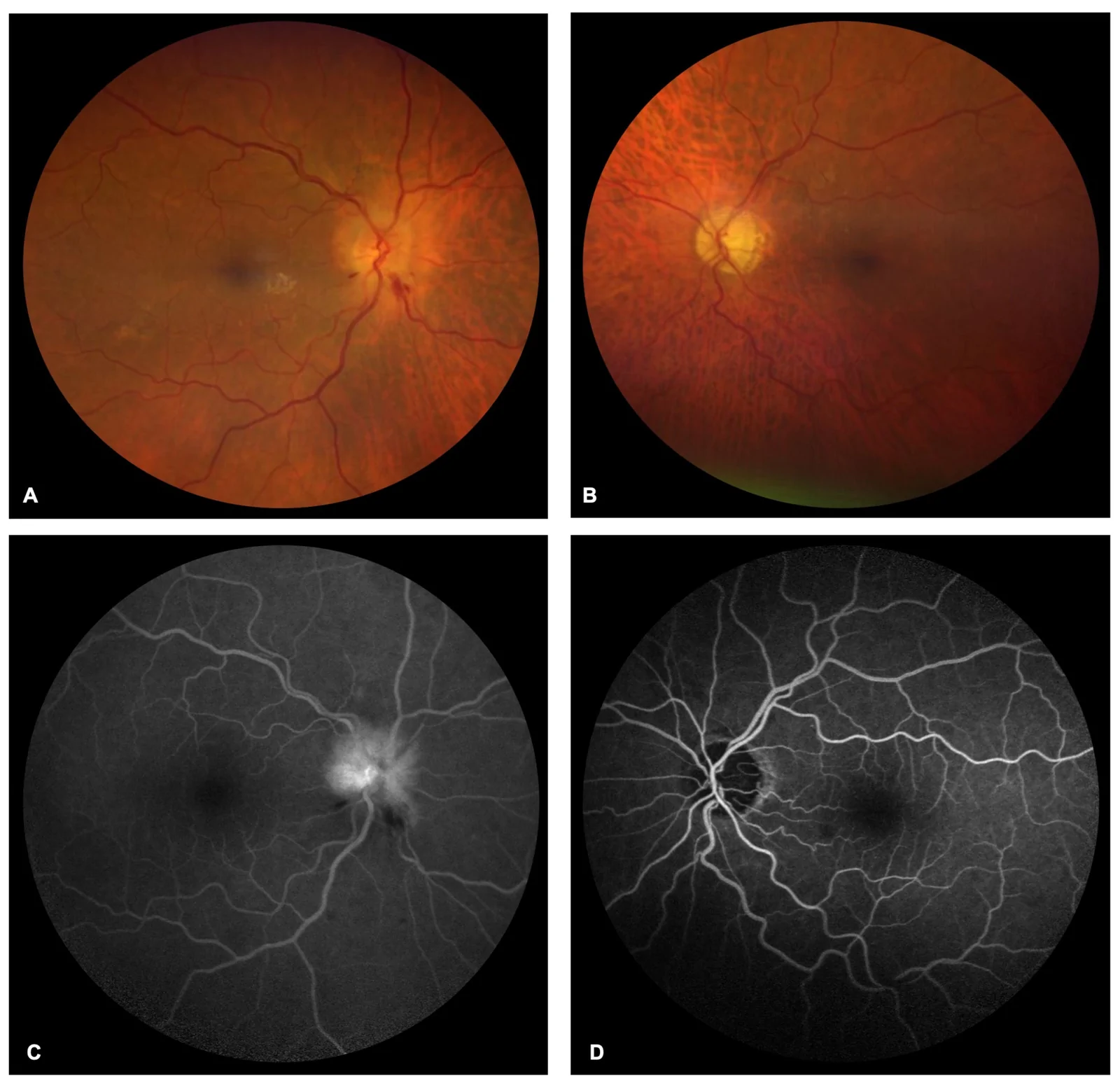
**Pseudo-Foster–Kennedy Syndrome.** (**A**,**B**) Color fundus photography (CFP) shows optic disc swelling with indistinct margins associated with peripapillary flamed hemorrhages in the right eye (RE) and pale and atrophic optic disc in the left eye (LE). (**C**,**D**) Fluorescein angiography shows hot disc in RE and hypofluorescent atrophic optic disc in LE.

FA can be used for the evaluation of ODE, masking the choroidal fluorescence in the peri-papillary zone during the arterial phase and showing optic disc leakage and blurry margins in the late phase of the exam (Figure 5) [36]. This last feature can be distinctive in the differential diagnosis between ODE and ODD, since no leakage is detected in pseudopapilledema, while it is always evident in true papilledema cases. Additionally, in case of increased ICP, the choroidal fold along the ONH border is detectable by FA. This finding can appear as a thin hypofluorescent line with hyperfluorescent borders, in case of superficial folding, or as a line with increased or decreased fluorescence in case of complete choroidal folding [37]. The choroidal fold is often detected by different imaging modalities, while it can escape fundus examination.

In ODE (e.g., papilledema or optic neuritis), RNFL appears thickened due to axoplasmic stasis in the acute phase, while in the chronic phase, optic atrophy manifests with RNFL thinning, often sectoral and consistent with previous edema distribution [22,32].

OCTA is also useful in cases of ODE, since the RPC network may appear dilated and tortuous, or reduced in visibility, depending on RNFL swelling or atrophic status [38]. OCTA aids in differentiating true edema from pseudopapilledema and in monitoring conditions such as hypertensive and diabetic optic neuropathies.

In NAION and AAION eyes, OCTA shows reduced VD and decreased vascular tortuosity in affected sectors. These changes, more pronounced in AAION, are often seen in the superior and temporal RPC zones. Sectoral choroidal dropout may also be visible, and quantitative differences in VD and tortuosity provide prognostic information [39,40].

NAION has been associated to SARS-CoV-2 infection. During the COVID pandemic, the availability of long and more demanding exams was drastically reduced. Therefore, the chance to use OCTA instead of FA provided a significant support to diagnose vascular diseases involving the retina and the optic disc (Figure S3) [41].

In case of ODE detected by fundus examination and CFP, OCT confirms the edema as a false increase in RNFL and could exclude a pseudopapilledema related to ODD. The images analysis combined with clinical presentation is crucial for making the correct diagnosis and guiding patients toward further tests and appropriate therapy. Beyond verifying the ODE, FA could provide support in the differential diagnostic pathway. In the chronic phase, CFP and OCT represent the first-line tests to monitor patients properly.

## 6. Retinal Vascular Diseases

**Figure 6:**
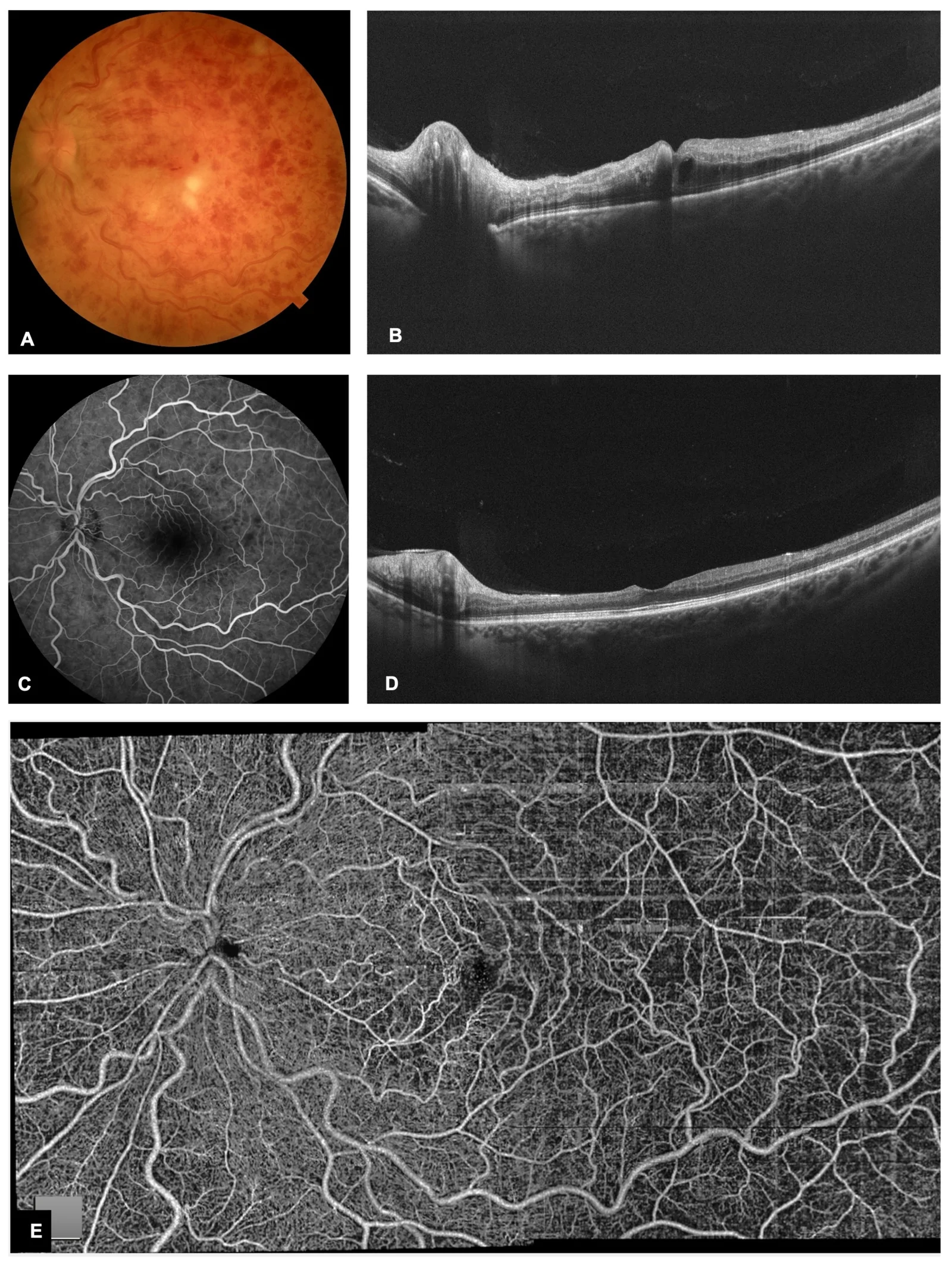
**Central retinal vein occlusion.** (**A**) Color fundus photography (CFP) of the left eye (LE) shows diffuse hemorrhages involving the posterior pole and beyond the arcade, slight optic disc swelling with indistinct margin. (**B**) Optical coherence tomography (OCT) shows few intraretinal hyporeflective cystic spaces and intraretinal hemorrhage. (**C**) Fluorescein angiography shows vascular tortuosity and diffuse hypofluorescent spots corresponding to retinal hemorrhages. (**D**) OCT performed after 3 intravitreal injections of Aflibercept 2mg shows the significant decrease in retinal swelling and cystoid macular edema. (**E**) OCT angiography highlights few areas of absent flow inferiorly to the macula with its involvement and in the temporal sector.

Fundus examination and CFP are also fundamental for the diagnosis of retinal vascular diseases which involve the optic disc, such as retinal vein occlusion and proliferative diabetic retinopathy (Figure 6). In the former, CFP allows monitoring the evolution over time of the ODE and hemorrhages and assessing the development of optic disc collaterals after resolution of the acute phase. In the latter, the CFP evaluation is useful to detect and monitor the onset of optic disc neovascularization (NVD), which could also be confirmed by FA and OCTA.

**Figure 7:**
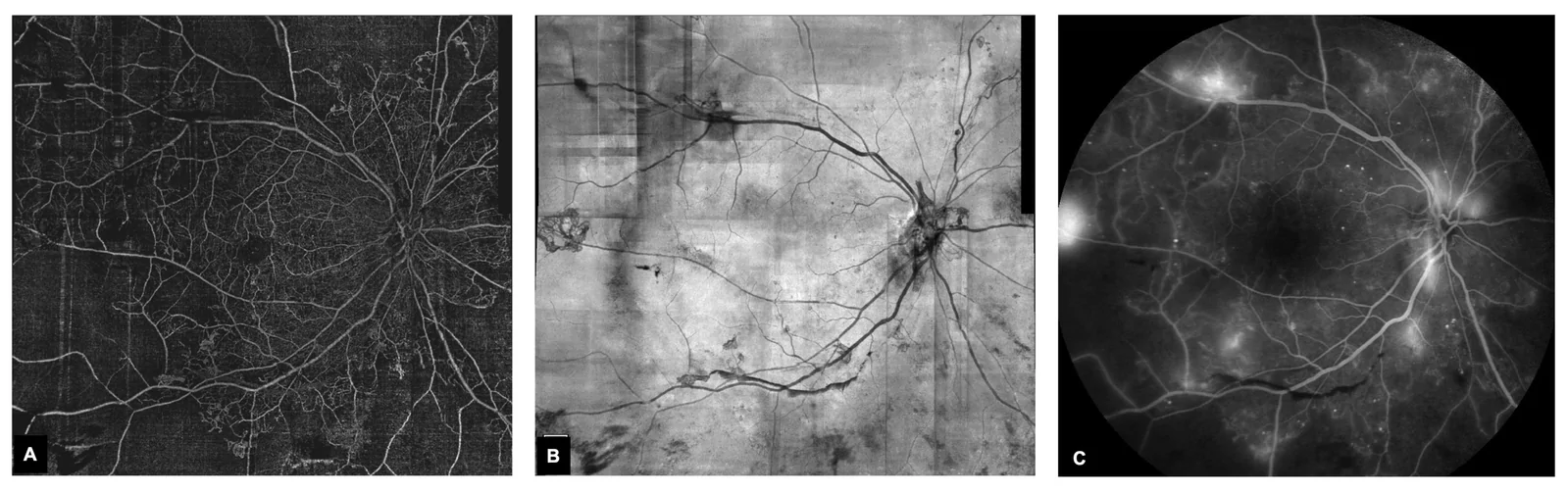
**Proliferative diabetic retinopathy (PDR) with optic disc neovascularization (NVD).** (**A**) Optical coherence tomography angiography (OCTA) shows areas of no flow surrounding and involving the posterior pole, with increased foveal avascular zone (FAZ) area. Additionally, the interruption of capillary vessels is highlighted at the watershed area. (**B**) The enface OCT highlights the presence of NVD and neovascularization elsewhere (NVE), which result in hyperfluorescence with dye leakage in fluorescein angiography (FA) (C). (**C**) FA clearly shows the ischemic areas at posterior pole and in the middle periphery in association with microaneurysms and hypofluorescent masked linear finding along the inferior vascular arcade corresponding to pre-retinal hemorrhage.

FA is essential to demonstrate the presence of NVD, which typically leaks during the exam, and to differentiate them from optic disc collaterals that differently do not present any leakage (Figure 7). Nowadays, the structural evaluation of retinal and disc neovascularization could be obtained by OCTA, which could only partially substitute FA, but OCTA may be useful whereas FA is not available or cannot be performed.

## 7. Multiple Sclerosis

In multiple sclerosis (MS), GCIPL thickness has been demonstrated as a useful predictor of the disease. A significant decrease in average RNFL, GCIPL and peripapillary VD has been reported in patients with MS, especially in case of lesion with high load in T2 in MRI. Moreover, changing in these biomarkers has been observed to correlate with reactivation of the disease [42]. However, OCT has some limitations. Particularly, evidence is reported in high-myopia patients, due to segmentation errors and compressive tilting of the ONH, affecting RNFL measurements and resulting in false-positive readings [43]. Furthermore, it is fundamental to highlight that normal datasets are not yet available for patients aged < 18 years. OCT remains an important tool for structural assessment in neuro-ophthalmology, enabling both diagnosis and longitudinal monitoring of the diseases. Nowadays, its reproducibility and availability make it a gold standard in optic nerve evaluation.

In optic neuritis, especially in MS, peripapillary VD is reduced even in eyes without a clinical history of neuritis, indicating the presence of subclinical damage [44]. Similarly, OCTA has been investigated in neurodegenerative diseases such as Alzheimer’s disease, in which macular thinning and reduced VD correlate with cognitive decline [45].

## 8. Inflammatory Diseases

Ocular sarcoidosis typically appears as bilateral anterior uveitis, although all segments may be involved [46]. Neurosarcoidosis is a rare and severe presentation that could show as an optic neuritis, optic disc granuloma or even papilledema due to central nervous system (CNS) involvement. In case of ONH granuloma, ICGA shows optic disc hypercyanescence and the OCT B scan confirms the presence of granuloma, which appears as a nodular hyperreflective lesion without evidence of optic disc cupping or retinal layer distinctions. Subretinal and intraretinal fluid may be associated. Following the treatment, the lesion size decreases with consequent reduction in ICGA hypercyanescence [47,48].

Tuberculosis can also be responsible of ONH granuloma, known as tuberculoma. However, optic disc involvement in tuberculosis may present different scenarios such as papillitis, ODE, retrobulbar neuritis or neuroretinitis. Few cases of compressive optic neuropathy and AION were also reported [49,50].

In case of optic disc involvement in Behçet disease, papillitis in the active phase or secondary optic disc atrophy related to previous ischemic vasculitis could be appreciated.

Beyond the typical retino-choroidal manifestations in Vogt–Koyanagi–Harada (VKH) disease, the ONH may be involved. The optic disc appears hyperemic and edematous, with early uniform hyperfluorescence and late leakage in FA, while no leakage is evident in ICGA [51].

## 9. Conclusions

Multimodal imaging has completely revolutionized each ophthalmic field, allowing for more detailed evaluation of pathological conditions, detection of novel biomarkers, new diagnoses and more precise analysis of disease progression. No single imaging modality is sufficient for all optic disc disorders.

Fundus examination and the related CFP still represent the milestone to start the diagnostic pathway. The pale appearance of an edematous optic disc may suggest an arteritic nature of AION, more than a non-arteritic base. As well, increased optic disc cupping, DHs and reduced neural rim highly raise the suspicion of glaucoma.

The introduction of OCT has mainly revolutionized the analysis of ocular diseases. Regarding the optic disc, the chance to quantitatively analyze the RNFL and GCIPL has allowed for an early diagnosis and more rigorous follow-up of patients suffering from glaucoma. Additionally, structural OCT provides detailed analysis of optic disc architecture and better evaluation of different conditions such as ODD, ODE and optic pit.

Among the angiographic exams, FA plays a significant role in the interpretation of optic disc conditions, highlighting the optic disc leakage in case of AION, which is not evident in case of ODD, as well as the NVD leakage in vascular retinal diseases. Although the most recently introduced OCTA is still the subject of study, it has already demonstrated its potential utility in the characterization of RPC plexus in both vascular and neurodegenerative diseases.

Table 1 summarizes the optic disc characteristics by different imaging modalities in the above-described conditions.

**Figure 8:**
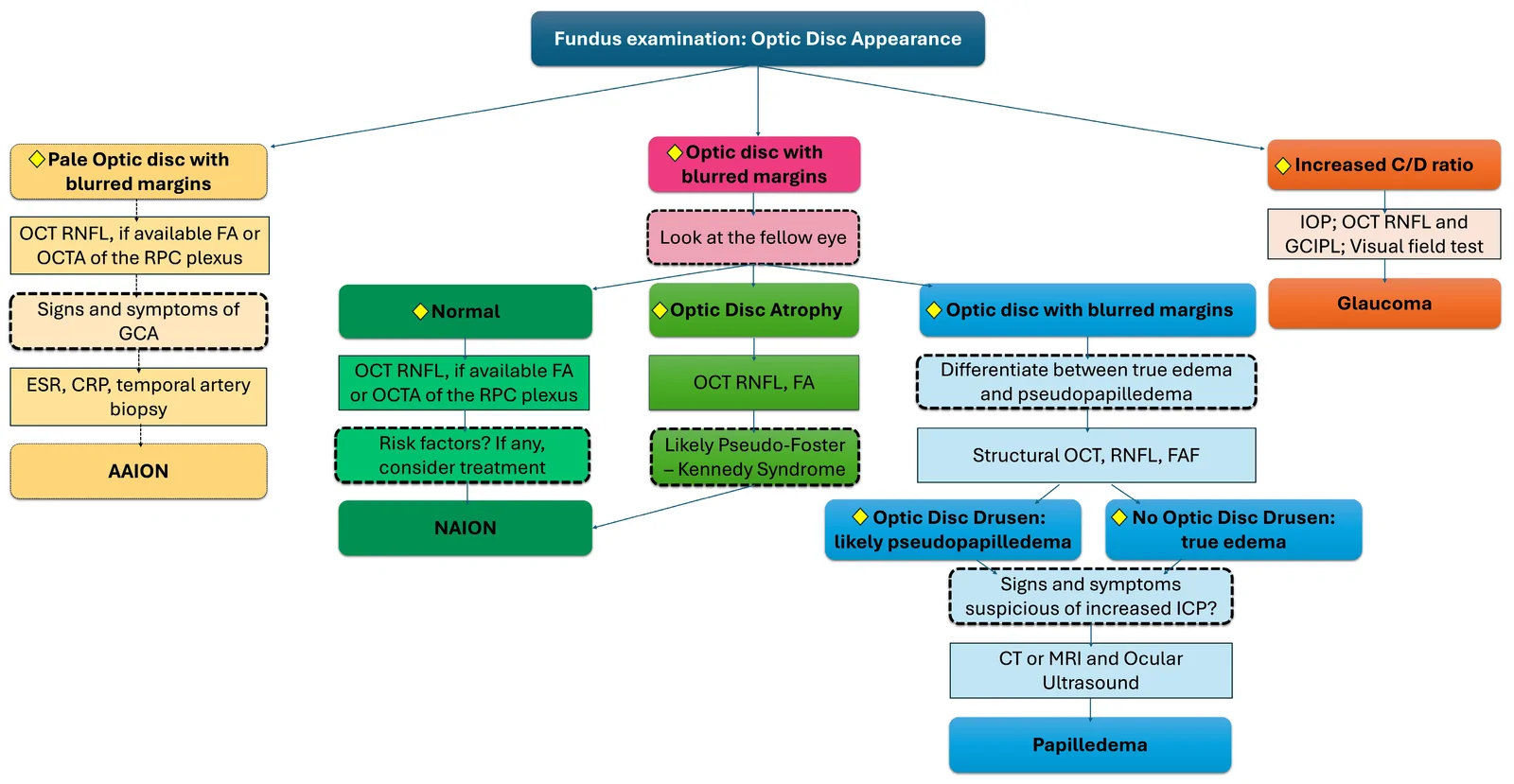
**Diagnostic workflow.** The workflow highlights the step-by-step diagnostic pathway in case of anomalous appearance of the optic nerve head at fundus examination. Colors were used to differentiate between different optic disc aspects and diseases. **Abbreviations:** OCT: optical coherence tomography; RNFL: retinal nerve fiber layer; FA: fluorescein angiography; OCTA: optical coherence tomography angiography; RPC: radial peripapillary capillary; GCA: giant cell arteritis; ESR: Erythrocyte Sedimentation Rate; CRP: C-Reactive Protein; AAION: arteritic anterior ischemic optic neuropathy; NAION: non-arteritic anterior ischemic optic neuropathy; FAF: fundus autofluorescence; ICP: intracranial pressure; CT: computed tomography; MRI: magnetic resonance imaging; C/D: cup-to-disc; IOP: intraocular pressure; GCIPL: ganglion cell–inner plexiform layer.

Figure 8 shows the diagnostic workflow to use in case of anomalous optic disc appearance.

In the imaging era, it is fundamental to highlight that each exam should be interpreted in light of the patient’s anamnesis, referred symptoms, and clinical findings to make the correct diagnosis.

Multimodal imaging plays a pivotal role in the neuro-ophthalmology field, offering unparalleled insight into ONH architecture and vascular integrity in both normal and diseased states. It enhances diagnostic precision, facilitates early detection of pathology, and supports individualized patient management across a spectrum of different optic nerve conditions [34].

Beyond diagnosis, multimodal imaging is increasingly used to monitor treatment response. Structural and vascular biomarkers, obtained through OCT, OCTA and FA, can document disease evolution before and after therapeutic interventions, providing objective evidence of treatment efficacy in inflammatory, ischemic and neurodegenerative optic neuropathies [52,53,54,55,56,57,58].

The future of optic disc imaging will likely rely on integrated structural, vascular and AI-assisted biomarkers rather than isolated imaging findings.

## 10. Methods of Literature Research

This narrative review was based on a non-systematic literature search performed in PubMed between September 2024 and September 2025. The search combined Medical Subject Headings (MeSH) and free-text terms related to optic disc disorders (including optic neuritis, ischemic optic neuropathy, papilledema, optic disc drusen, glaucoma, and hereditary or compressive optic neuropathies) with terms related to multimodal retinal and optic nerve imaging (including OCT, OCTA, FA, ICGA, FAF and CFP). Articles published in English between 2006 and 2025 were considered.

Original studies, systematic reviews, meta-analyses, narrative reviews, and large case series were evaluated. Priority was given to studies with robust methodology, larger sample sizes, and findings considered clinically relevant to the diagnosis and imaging characterization of optic nerve disorders. Titles and abstracts were initially screened, followed by full-text review of articles considered relevant. Additional references were identified through manual screening of the bibliographies of selected publications when appropriate.

As this article was designed as a narrative review intended to provide a clinically oriented synthesis of current evidence, no formal systematic review methodology, predefined inclusion/exclusion criteria, risk-of-bias assessment, or evidence grading system was applied.

## Figures and Tables

**Table 1:** **Optic disc appearance by different imaging modalities in optic nerve diseases.** A summary of the optic disc appearance in glaucoma, optic disc drusen, papilledema, non-arteritic and arteritic anterior ischemic optic neuropathy is reported. A color-coding system is assigned to distinguish between first-line tests (green), confirmatory tests (blue), complementary tests (yellow) and tests required only in selected cases (orange).

Disease	CFP	FAF	OCT	OCTA	FA
**Glaucoma**	Increased cupping Thinning/notching of neuroretinal rim Vertical cup elongation Vessel bayoneting Β-zone peripapillary atrophy Disc hemorrhages	-	Decreased RNFL and GCC	Focal or diffuse decrease in RPC plexus vessel density Decreased perfusion density	No leakage
**Optic Disc Drusen (ODD)**	Superficial ODD: Yellowish, highly reflective bodies Crowded optic disc Deep ODD: Non-visible Undefined disc margins.	Superficial ODD: Hyperautofluorescent Deep drusen (>500 µm below the ILM): Non-visible	Thinning RNFL Rounded hyporeflective core with hyperreflective border	Dilated RPC plexus Tortuous RPC plexus	No leakage
**Papilledema**	Swollen, elevated optic disc Blurred optic disc margins Venous dilation Peripapillary hemorrhages Exudates Cotton wool spots	Indistinct hypoautofluorescent margins of the optic disc	Falsely increased RNFL in acute phase RNFL thinning when atrophy develops	Dilated RPC plexus	Optic disc leakage Blurred optic disc margins Hyperfluorescent choroidal folds
**NAION**	Sectoral disc edema (more commonly) in acute phase Optic disc atrophy in chronic phase	Indistinct hypoautofluorescent optic disc margins	Falsely increased RNFL in acute phase RNFL thinning when atrophy develops	Decreased RPC plexus vessel density Vascular tortuosity	Optic disc leakage Blurred optic disc margins
**AAION**	Pale optic disc Diffuse disc edema in acute phase Optic disc atrophy in chronic phase	Indistinct hypoautofluorescent margins of the optic disc	Falsely increased RNFL in acute phase RNFL thinning when atrophy develops	Decreased RPC plexus vessel density Vascular tortuosity	Optic disc leakage Blurred optic disc margins Delayed choroidal filling

**Abbreviations:** CFP: color fundus photography; FAF: fundus autofluorescence; OCT: optical coherence tomography; OCTA: OCT angiography; FA: fluorescein angiography; ODD: optic disc drusen; NAION: non-arteritic anterior ischemic optic neuropathy; AAION: arteritic anterior ischemic optic neuropathy; RNFL: retinal nerve fiber layer; GCC: ganglion cell complex; RPC: radial peripapillary capillary.

## Data Availability

No new data were created or analysed in this study. Data sharing is not applicable to this article.

## Supplementary Materials

The following supporting information can be downloaded at: https://www.mdpi.com/article/10.3390/diagnostics16162591/s1, Figure S1. Multimodal imaging of bilateral healthy optic disc. Figure S2. Advanced glaucomatous optic disc. Figure S3. Non-arteritic anterior ischemic optic neuropathy (NAION) in a SARS–CoV–2 patient.

## Abbreviations

ONH: optic nerve head; OCT: optical coherence tomography; EDI: enhanced depth imaging; OCTA: optical coherence tomography angiography; FAF: fundus autofluorescence; PPA: peripapillary atrophy; PHOMS: peripapillary hyperreflective ovoid mass-like structures; ODD: optic disc drusen; NAION: non-arteritic anterior ischemic optic neuropathy; RNFL: retinal nerve fiber layer; CFP: color fundus photography; ICP: intracranial pressure; FA: fluorescein angiography; RPE: retinal pigment epithelium; ILM: inner limiting membrane; ODE: optic disc edema; MRI: magnetic resonance imaging; AION: anterior ischemic optic neuropathy; AAION: anterior ischemic optic neuropathy; MS: multiple sclerosis; RPC: radial peripapillary capillary; VD: vessel density; PD: perfusion density.
