# Supplementary material for: Diagnostic Role of Multimodal Imaging in Optic Disc Diseases
Source: Diagnostics (Basel). 2026 Aug 16;16(16):2591. doi: 10.3390/diagnostics16162591 (PMC13512194; doi:10.3390/diagnostics16162591)
Supplement: Supplementary file 1 [file diagnostics-16-02591-s001.zip › diagnostics-4483177-supplementary.pdf]

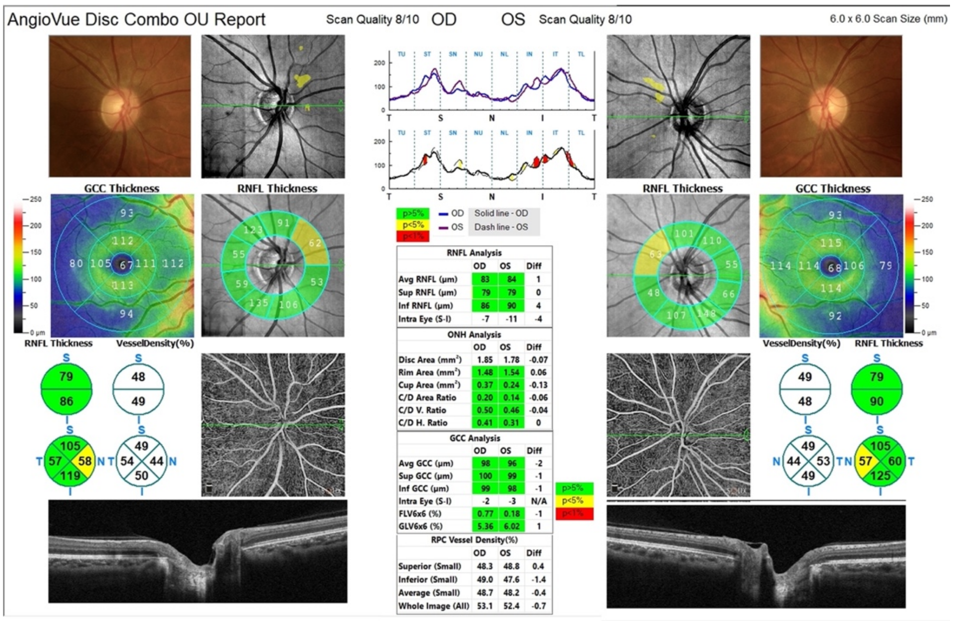

Figure S1. Multimodal imaging of bilateral healthy optic disc. Multimodal imaging of healthy optic discs, including colour fundus photography and infrared images, the ganglion cellular complex (GCC) and retinal nerve fibre layer (RNFL) thickness map. Optical coherence tomography (OCT) angiography (OCTA) of the optic nerve head (ONH) at radial peripapillary capillary (RPC) plexus and corresponding vessel density are reported. Horizontal OCT B scans centred on the optic discs cupping are evident at the bottom. In the middle, quantitative data are reported.

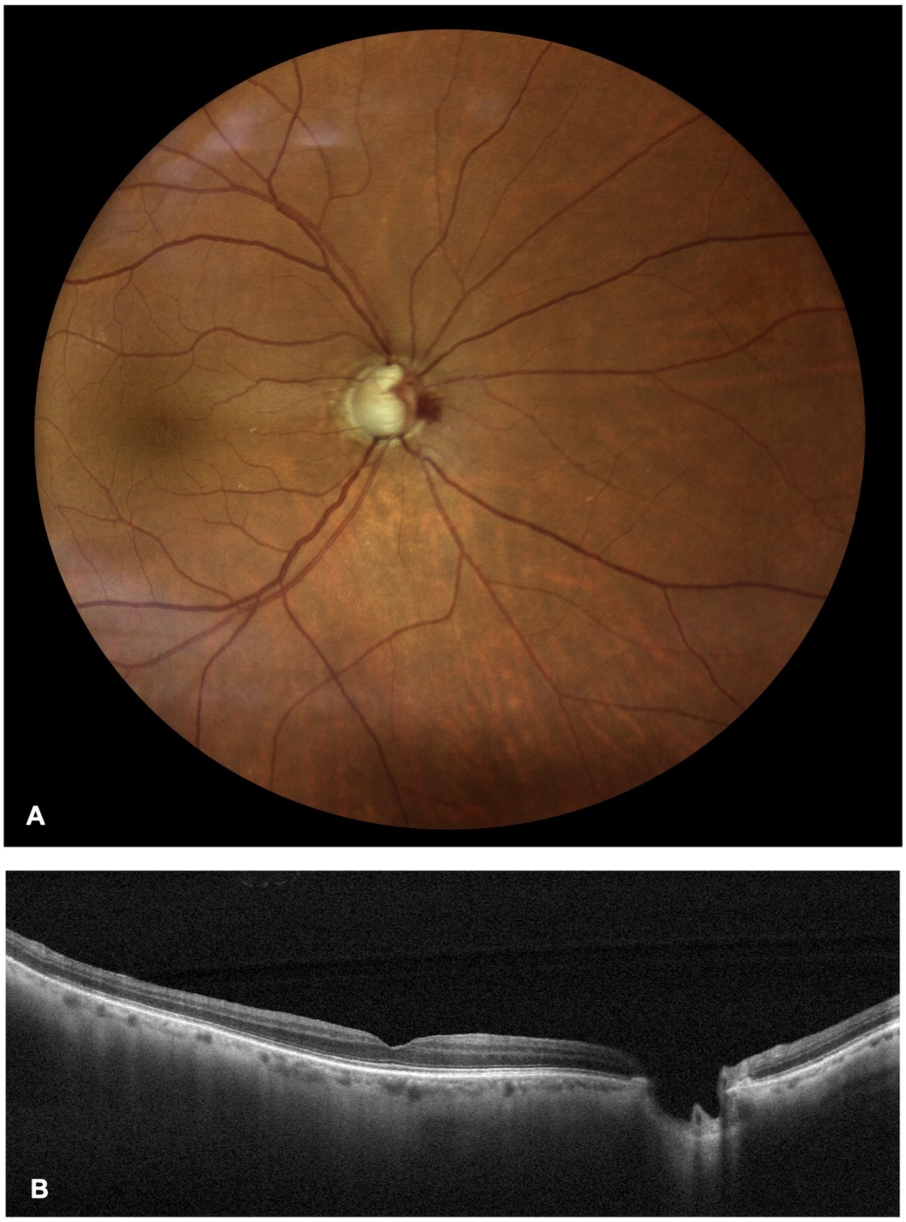

Figure S2. Advanced glaucomatous optic disc. (A) The optic disc appears with defined margins, significant increased cupping and remarkable reduction in the neural rim and a split haemorrhage nasally. (B) The optical coherence tomography (OCT) B scan highlights the increased cupping and the reduced retinal nerve fibre layer (RNFL), while the other retinal layers are evident.

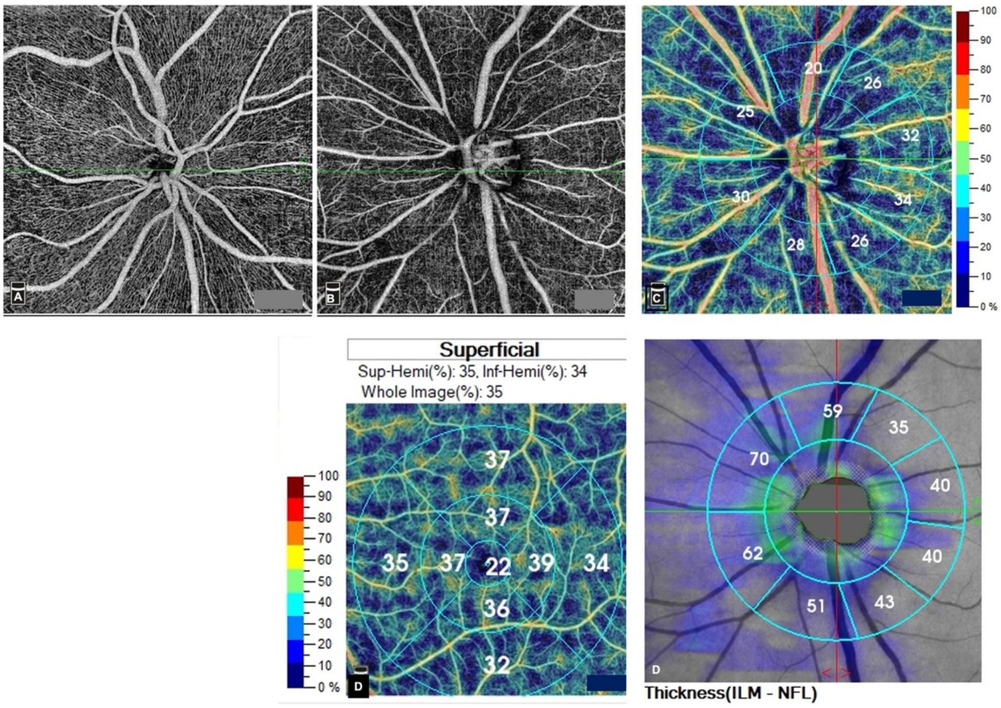

Figure S3. Non-arteritic anterior ischemic optic neuropathy (NAION) in a SARS–CoV–2 patient. (A) Optical coherence tomography angiography (OCTA) of the right eye (RE) shows preserved radial peripapillary capillary (RPC) plexus. (B) The OCTA of the left eye (LE) shows area of no flow involving the RPC, as confirmed by the vessel density (VD) map (C), which shows the area of non perfusion in blue. (D) The retinal VD of LE shows its significant decrease in the superficial capillary plexus highlighting the remarkable ischemic phenomenon. (E) The thickness map of the optic disc of LE shows the decrease in retinal nerve fibre layer (RNFL) involving all the quadrants.
